# “What’s Your Taste in Music?” A Comparison of the Effectiveness of Various Soundscapes in Evoking Specific Tastes

**DOI:** 10.1177/2041669515622001

**Published:** 2015-12-28

**Authors:** Qian (Janice) Wang, Andy T. Woods, Charles Spence

**Affiliations:** Department of Experimental Psychology, Oxford University, UK; Crossmodal Research Laboratory, Oxford University, UK; Department of Experimental Psychology, Oxford University, UK

**Keywords:** Crossmodal correspondences, Internet-based testing, soundtracks, taste, emotion mediation

## Abstract

We report on the results of two online experiments designed to compare different soundtracks that had been composed (by various researchers and sound designers) in order to evoke/match different basic tastes. In Experiment 1, 100 participants listened to samples from 24 soundtracks and chose the taste (sweet, sour, salty, or bitter) that best matched each sample. Overall, the sweet soundtracks most effectively evoked the taste intended by the composer (participants chose *sweet* 56.9% of the time for the sweet soundtracks), whereas the bitter soundtracks were the least effective (participants chose *bitter* 31.4% of the time for the bitter soundtracks), compared with chance (choosing any specific taste 25% of the time). In Experiment 2, 50 participants rated their emotional responses (in terms of pleasantness and arousal) to the same 24 soundtrack samples and also to imaginary sweet/sour/salty/bitter-tasting foods. Associations between soundtracks and tastes were partly mediated by pleasantness for the sweet and bitter tastes and partly by arousal for the sour tastes. These results demonstrate how emotion mediation may be an additional mechanism behind sound-taste correspondences.

## Introduction

In recent years, a growing body of empirical research has started to emerge demonstrating the intriguing relationship between what we hear and what we taste. Naturally, part of this relationship involves how the sounds of consumption affect the experience of eating, for example, amplifying food crunching sounds has been shown to increase the perception of crispiness of crisps (Zampini & Spence, 2004; see also Demattè et al., 2014, and Spence, 2015, for a recent review). Another such area involves background noise and its impact on taste perception (Spence, Michel, & Smith, 2014). For example, white noise mimicking the conditions inside an airline cabin has been shown to selectively reduce the perceived intensity of sweet tastes (Yan & Dando, 2015). Meanwhile, performing a vocal shadowing plus musical distraction task can significantly impair participants’ ability to discriminate the alcohol content of a drink (Stafford, Agobiani, & Fernandes, 2013; see Spence, 2014, for a review).

Moving beyond environmental sounds and their impact on taste/texture/flavor perception, one also has to consider the influence of crossmodal correspondences. These are the general associations that people make between seemingly unrelated attributes (or dimensions) of different sensory modalities (see Spence, 2011, for a review). A growing list of correspondences have now been documented between specific sound and taste attributes—such as, for example, between high pitch and sourness, or between low pitch and bitterness (e.g., Crisinel & Spence, 2009, 2010; Mesz, Trevisan, & Sigman, 2011; see Knöferle & Spence, 2012, for a review).

As part of this growing movement to study sound-taste correspondences, a number of researchers and sound designers have recently started to compose their own soundtracks to match (or, in some sense, go together well with) specific tastes. Increasingly, such soundscapes/soundtracks are being used in both scientific research and artistic performances (e.g., Crisinel et al., 2012; Knoeferle, Woods, Käppler, & Spence, 2015; Kontukoski et al., 2015; Mesz, Sigman, & Trevisan, 2012; Reinoso Carvalho, Van Ee, Rychtarikova, Touhafi, Steenhaut, Persoone, & Spence, 2015; Reinoso Carvalho, Van Ee, Rychtarikova, Touhafi, Steenhaut, Persoone, Spence, & Leman, 2015; Wang, 2013). Such soundtracks could have either or both of the following aims: (a) to demonstrate a connection between taste and sound in terms of participants being able to associate certain soundtracks with specific tastes and (b) to alter the perceived taste of food and drink while participants listen to such soundtracks.

While the connection between soundscapes and tastes (or taste words) can initially seem surprising, it can also be seen as an extension of the power of music to express abstract ideas. Many years ago, when Rigg (1937) tested how well a group of college students could interpret the intended meanings of musical compositions, the participants could only reliably distinguish between broad categories of joyful and sad music but were unable to discriminate between more specific categories like *farewell* or *mourning*. More recently, however, Watt and Quinn (2007) have shown that music can be reliably associated with many higher level concepts, such as gender (i.e., male vs. female) and age (young vs. old). The fact that certain soundtracks can be reliably matched to particular taste words (e.g., Knoeferle et al., 2015; Mesz et al., 2012) demonstrates the strength of the crossmodal correspondences that exist between sound/music and taste.

One potential explanation for such matching of sound/music with taste is emotion mediation. That is, certain crossmodal correspondences may be explained by the common emotional associations (such as pleasantness or arousal, see Collier, 1996, for a reduction of emotion space down to two dimensions) of the various different stimuli involved. In general, such a hedonic matching account between seemingly unrelated stimuli presented in different sensory modalities has been suggested by a variety of sources (see e.g., Palmer, Schloss, Xu, & Prado-León, 2013; Schifferstein & Tanudjaja, 2004; Velasco, Woods, Deroy, et al., 2015; Velasco, Woods, Liu, et al., 2016). Relevant evidence pertaining to the case of crossmodal correspondences between audition and taste has, however, been limited to the pleasantness account. In an experiment designed to test for any crossmodal mappings between differently flavored chocolates (milk, marzipan, and dark) and sounds varying in their pitch and timbre (i.e., by instrument type), Crisinel and Spence (2012) reported that while their participants’ choice of instrument could be predicted by the pleasantness ratings they gave to the various chocolates, their choice of pitch was not. Besides pleasantness, to date, no one has looked at the potential role of emotional arousal levels in crossmodal correspondences between audition and taste.

Up until now, the soundtracks produced by various researchers and designers have never been tested in the same setting. Furthermore, some of the soundtracks, such as those used in Knoeferle et al. (2015) and Mesz et al. (2012), have only previously been tested in a constrained context in which the participants were essentially given a four-alternative-forced-choice task (i.e., with four soundtracks to listen to and four taste words to choose from). Therefore, in Experiment 1, we tested a group of these recently created taste-inspired soundtracks together in the same sound and taste word matching task. The participants were free to choose any taste word they wished for each of the soundtracks, in order to determine which of them exhibited the strongest association with a given taste. In Experiment 2, we used the same soundtracks in order to measure the role of emotion as a possible mediator in the soundtrack-taste matching task. We did not ask participants to match the soundtracks to taste words but instead asked for their emotion ratings (for pleasantness and arousal) of the soundtracks and of bitter/salty/sour/sweet-tasting foods. These emotional ratings were then combined with the soundtrack-taste matching results from Experiment 1 in order to calculate the potential role of pleasantness and arousal in mediating the crossmodal correspondence between audition and taste.

## Experiment 1

### Methods

#### Participants

One hundred participants (51 women, 49 men) aged between 21 and 62 years (*M* = 34.06, *SD* = 9.53) took part in the study. The participants gave their informed consent and reported no cold or other impairment of their senses of smell, taste, or hearing. The participants were recruited from Amazon’s Mechanical Turk. The experiment was approved by the central university research ethics committee of Oxford University (MSD-IDREC-C1-2014-205).

#### Auditory stimuli

Twenty-four root-mean-square normalized soundtracks (McCarthy, 2007) were used (five bitter, five salty, seven sour, and seven sweet). The soundtracks varied originally from 30 seconds to 6 minutes in length. Since the soundtracks were generally uniform in texture, we decided to use only the first 15 seconds of each soundtrack in order to have uniform length auditory stimuli and to limit the overall length of the experiment. Here is a brief description of the motivation and origins of the soundtracks, categorized by composer/designer (in alphabetical order):

**Condiment Junkie**: A UK sound branding agency recently released an EP album of taste soundtracks. We used excerpts of the sweet, sour, salty, and bitter soundtracks from the album. Note that an earlier version of the sweet and bitter soundtracks had been used previously in a study by Crisinel et al. (2012). There, the participants rated samples of bittersweet toffee on a 7-point bitter-sweet scale while listening to the two sounds. Their results revealed that the participants rated the cinder toffee samples (which came from the same batch) significantly differently under the two auditory conditions. In the same study, the two soundtracks were pretested in a control experiment in order to ensure that the participants rated the soundtracks differently on a 1 to 9 bitter-sweet scale. As expected, the sweet soundtrack (*M* = 6.68, *SD* = 1.78) was rated as significantly sweeter on the bitter-sweet scale than the bitter soundtrack (*M* = 2.97, *SD* = 1.14).

**Jialing Deng and Harlin Sun**: Designed a set of sweet, bitter, salty, sour, and umami soundtracks for synesthetic appetizer, part of Deng’s Masters of Arts Thesis project (June, 2015). The stated aim was to create a narrative environment of a synesthetic world by offering augmented eating experiences through crossmodal interactions. The soundtracks were designed to evoke specific tastes, with the goal of helping those who suffer from some form of sensory dysfunction and who might not otherwise be able to taste normally.

**Evan Kassof**: Designed sounds to match each of the four basic tastes. These sounds were used in a citizen science experiment as part of the science museum cravings exhibit in London, UK. The participants could either access the experiment at the gallery or online, via the science museum’s homepage or the cravings exhibition information page (http://www.sciencemuseum.org.uk/visitmuseum/Plan_your_visit/exhibitions/cravings/cravings-experiment.aspx). The sounds were composed from four basis soundtracks that, when combined in different ratios, created composite soundtracks that varied in terms of their articulation, pitch, loudness, and consonance (see Knöferle & Spence, 2012, Table 1). In the science museum experiment, the participants were presented with individual sounds and had to match them to a taste word (sweet, bitter, sour, salty, umami, or else indicated that they were *unsure*).

**Table 1.** Acoustic Properties of Each Soundtrack, Grouped by Taste.

Tastes	Composer / experimenter	Pitch	Instrument	Harmony	Articulation
Bitter	Condiment Junkie	low	synthesized wave functions and trombone	consonant	legato (rumble)
	Kassof	medium	cello, synthesized clicking sound	dissonant	legato (rumble)
	Reinoso Carvalho	medium	synthesized wave function	consonant	staccato
	Deng	low	synthesized bass	consonant	legato
	Knoeferle	low	synthesizer bass, pads, piano	dissonant	legato
Salty	Mesz	medium	marimba, percussion	consonant	staccato
	Condiment Junkie	medium	brass, salt shaker	dissonant	staccato
	Kassof	medium	cello, synthesized clicking sound	dissonant	staccato
	Deng	medium	salt shaker, tearing paper, bass, brass	dissonant	staccato
	Knoeferle	low	synthesizer bass, pads, piano	consonant	staccato
Sour	Mesz–Hapan	high	piano, synthesized percussion	consonant	staccato
	Mesz–Tango	high	piccolo, clarinet	dissonant	staccato
	Condiment Junkie	high	brass, woodwind	dissonant	staccato
	Kassof	high	cello, synthesized clicking sound	dissonant	staccato
	Wang	high	synthesized	dissonant	legato
	Deng	high	drums, brass, bass	dissonant	staccato
	Knoeferle	high	synthesizer bass, pads, piano	dissonant	staccato
Sweet	Mesz–Makea	high	piano	dissonant	staccato
	Mesz–Beethoven	medium	strings	consonant	legato
	Condiment Junkie	high	synthesized piano reverb, wave functions	consonant	legato with chimes
	Kassof	high	cello, synthesized clicking sound	consonant	legato with clicks
	Reinoso Carvalho	high	synthesizer	consonant	legato
	Deng	high	bells, piano, synthesizer	consonant	legato with chimes
	Knoeferle	high	synthesizer bass, pads, piano	consonant	legato

**Klemens Knoeferle and Florian Kaüppler**: Designed a set of taste soundtracks (sweet, bitter, sour, and salty) inspired by Knoeferle et al.’s (2015, Experiment 1) study in which the participants had to match a number of auditory parameters (attack, discontinuity, pitch, roughness, sharpness, and speed) to basic taste words (bitter, sweet, salty, and sour). Based on these results, low-level properties of a 30-second piece of synthesized music were systematically manipulated in order to create soundtracks matching different tastes (Knoeferle et al., 2015, Experiment 2). These soundtracks were then tested online with participants from the United States and India in a matching task where all four soundtracks and four taste words were presented as a group. On average, the North American participants matched 1.75 sounds correctly while the Indian participants matched 1.38 sounds correctly (as compared with matching one sound correctly by chance). Importantly, both groups performed at a level that was significantly better than chance.

**Bruno Mesz**: A group of musicians were initially asked to improvise on a MIDI keyboard based on the taste words sweet, sour, bitter, and salty (Mesz et al., 2011). Based on those improvisations, different loudness, pitch, duration, and articulation features were extracted for each taste. Those features were then used to design an algorithm that automatically generated music of specific tastes by combining fragments of classical and popular music that matched the aforementioned auditory features (Mesz et al., 2012). Note here that Mesz also composes his own pieces based on the same principles and uses his compositions for both research and multisensory performances. Five pieces from Mesz were tested in the present study (see Table 2 for details on each soundtrack).

**Table 2.** Summary of Each Composer/Designer’s Soundtracks and Their Relationship to Research.

Composer/ designer	Taste soundtracks	Usage	Previous testing
Condiment Junkie	Bitter, sweet, sour, salty, *umami*	Featured in EP album. Earlier versions of the bitter and sweet soundtracks were used in Crisinel et al.’s (2012) study to test whether music can bias taste ratings of bittersweet toffee	Earlier versions of the bitter and sweet soundtracks were tested by Crisinel et al. (2012) in a control study. 31 participants rated these soundtracks as significantly different on a bitter-sweet scale
Deng	Bitter, sweet, sour, salty, *umami*	MA thesis on multisensory eating experiences	None
Kassof	Bitter, sweet, sour, salty	Used in study at science museum’s cravings exhibit. The participants had to match a basic taste word to each soundtrack. The soundtracks were presented independently and not all of the participants heard all of the soundtracks	Tested as part of science museum exhibit. No results as yet. The exhibit is currently on-going
Knoeferle	Bitter, sweet, sour, salty	Used in Knoeferle et al.’s (2015) study where participants from India and the United States assigned four basic taste words to the four soundtracks presented together	Tested by Knoeferle et al. (2015). On average, United States participants assigned 1.75 of the four sounds to the correct taste word, while the Indian participants assigned 1.38 sounds correctly. Both groups performed at a level that was better than chance
Mesz	Sweet, sour, salty	The sweet Makea soundtrack was used by Mesz et al. (2012). The sour tango piece was used by Kontukoski et al. (2015). The soundtracks have also been presented in a number of multisensory performances	The sweet Makea soundtrack appeared in Mesz et al. (2012), where it was tested by in a four-taste four-soundtrack matching task
Reinoso Carvalho	Bitter, sweet, *neutral*	Used in Reinoso Carvalho et al. (2015) to study how participants matched soundscapes to different chocolates, and how soundscapes influenced the taste ratings of chocolates	A control experiment was conducted in which 78 participants rated the soundtracks as significantly different on a bitter-sweet scale (the three soundtracks were presented together and the participant could compare between them)
Wang	Sour	Used to test perceptual effects of sweet, sour, and bitter music on drinks (Wang, 2013)	None

**Felipe Reinoso Carvalho**: In collaboration with the Institute for Psychoacoustics and Electronic Music at the University of Ghent, Reinoso Carvalho designed sweet and bitter soundtracks. The soundtracks were produced by Tim Vets @ IPEM, UGent; coproduced by Felipe Reinoso Carvalho, Sander de Keer, and Tomas Serrine; and mastered by Felipe Reinoso Carvalho at sonictaste.flavours.me (2013).

The bitter and sweet soundtracks (along with a neutral soundtrack which was not used in the present study) were used in a recent chocolate-tasting study (see Reinoso Carvalho, Van Ee, Rychtarikova, Touhafi, Steenhaut, Persoone, Spence, & Leman, 2015). These soundtracks were designed to be congruent with the taste of bitter and sweet chocolate, respectively. The soundtracks were inspired by Crisinel and Spence’s (2010; Crisinel et al., 2012) previous work.

The sounds were first tested in an online study by 78 participants, who rated the soundtracks as significantly different on a 6-point bitter-sweet scale. During the actual study, the participants first matched chocolate samples to the soundtracks and then tasted the samples while listening to the soundtracks.

**Qian (Janice) Wang**: Designed a sour soundtrack for a study on the effect of taste-congruent sounds on taste evaluation, as part of her masters’ degree at the MIT Media Lab (Wang, 2013). The soundtrack was composed in Ableton Live with features of high pitch, fast tempo, and high dissonance (Mesz et al., 2011). The soundtrack consisted of notes played by synthetic instruments. The pitch of the notes ranged from C2 to C6. During the study, the participants listened to bitter, sweet, and sour soundtracks (bitter and sweet soundtracks were the same as used in Crisinel et al., 2012) while rating juice samples in terms of their bitterness, sweetness, and sourness.

The soundtrack excerpts selected for use in the present study can all be heard at https://soundcloud.com/janicewang09/sets/taste-soundscapes-test. See Table 1 for a list of soundtracks and their descriptions, organized by taste; see Table 2 for a list of all soundtracks, their usages, and any previous tests of taste associations. Note that the italicized soundtracks were not used in the present study.

#### Procedure

The experiment was programmed on the Xperiment experiment-design and hosting platform.^1^ Before the actual study began, the participants specified their gender, age, country of origin, and self-rated their musical expertise levels (the choices were none, amateur, intermediate, or advanced). The participants had to listen to all 24 soundtracks in a random order. After each soundtrack, the participants had to choose which basic taste (sweet, sour, bitter, or salty) best matched the sound clip that they had just heard and rate how confident they were in having decoded the correct taste on a scale from 0 to 100. The presentation of taste choices was randomized for each trial.

The study lasted for approximately 10 minutes, and the participants were paid $1.20 USD for taking part in the study.

### Results

**Figure 1.**
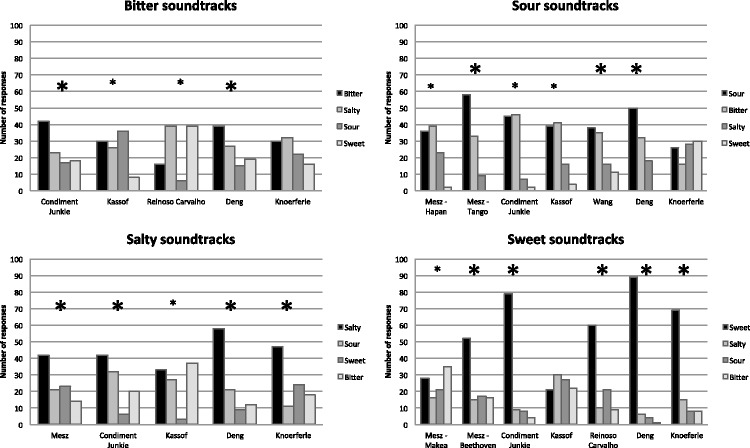
Results of participants’ soundtrack-taste matching organized by each taste that the soundtracks were designed to evoke in Experiment 1. The chart reveals the distribution of participants’ choice of taste matches for each soundtrack, with a total of 100 responses for each soundtrack. Soundtracks with nonrandom distributions of taste ratings (i.e., participants are not just matching tastes by chance) are shown with asterisks (*p* < .05); out of these, the soundtracks where the most chosen taste was also the taste intended by the composer are shown by larger asterisks.

The choices for the best-matching taste word were tallied for each soundtrack (see Figure 1 for chart and Appendix A for a table listing the precise values). The soundtracks with the highest rate of matching for each taste were as follows: for bitterness, Condiment Junkie (42% of participants matched it with bitter, and they were 1.82 times more likely to match it with bitter than with salty, the next most popular choice); for saltiness, Deng (58% of participants matched it with salty and were 2.76 times more likely to match it with salty than with sour, the next most popular choice); for sourness, Mesz–Tango (58% of participants matched it with sour and were 1.76 times more likely to match it with sour than with bitter, the next most popular choice); and for sweetness, Deng (89% of participants matched it with sweet and were 14.83 times more likely to match it with sweet than with salty, the next most popular choice). In comparison, had the participants been responding randomly, 25% of them would have been expected to match each taste (bitter, salty, sour, and sweet) to any given soundtrack.

**Figure 2.**
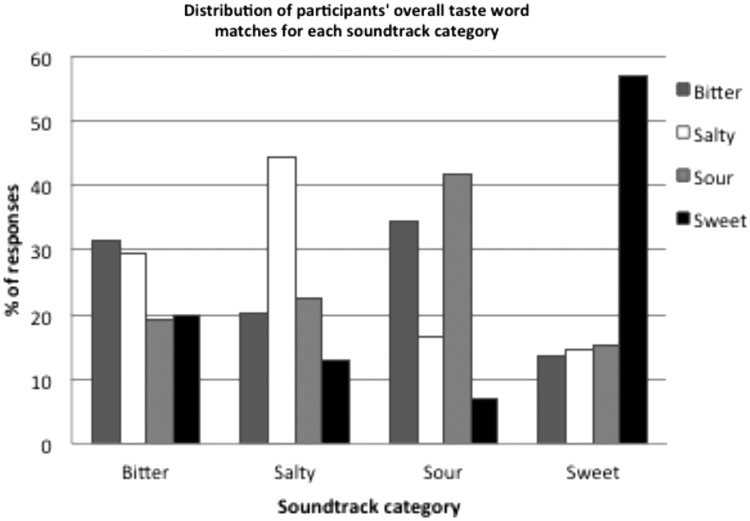
Overall distribution of participants’ taste word matches in Experiment 1, averaged over all soundtracks in each taste category.

Averaging over all of the soundtracks that were associated with each taste, 31.4% of the participants’ responses were correct for bitter soundtracks, 44.4% of participants’ responses were correct for salty soundtracks, 41.7% of the participants’ responses were correct for sour soundtracks, and 56.9% of participants’ responses were correct for sweet soundtracks (see Figure 2). A chi-square test for independence was conducted in order to assess whether different taste words were chosen for different taste soundtracks, tallied over all of the soundtracks that had been generated for each taste. The results indicated that the different taste soundtracks influenced the choice of taste words (χ^2^(9, 2400) = 720.62, *p* < .0001). The strength of this effect, measured by computing Cramer’s V, can be classified as medium (*V* = .32).

A chi-square test of goodness of fit was calculated for each soundtrack to determine which of them induced a distribution of taste matches that was significantly different from chance. In fact, out of the 24 soundtracks, only three had nonsignificant preferences in the choice of taste matches (Knoeferle-bitter, Knoeferle-sour, Kassof-sweet). The resulting chi-square values can be seen in Table 3. Out of the 21 soundtracks with nonrandom distributions of responses, for 14 of them, the most chosen taste word was also the one intended by the composer (see Figure 1).

**Table 3.** Results of Chi-Square Test of Good of Fit for Participant’s Choice of Taste Word Matches for Each Soundtrack in Experiment 1, Grouped by Taste Category.

Tastes	Composer/designer	χ^2^(3,100)	*p*
Bitter	Condiment Junkie	16.24	.001
	Kassof	17.44	.001
	Reinoso Carvalho	33.36	<.0005
	Deng	13.44	.004
	Knoeferle	6.56	.087
Salty	Mesz	17.20	.001
	Condiment Junkie	28.96	<.0005
	Kassof	27.84	<.0005
	Deng	61.2	<.0005
	Knoeferle	29.2	<.0005
Sour	Mesz–Hapan	34.00	<.0005
	Mesz–Tango	36.02	<.0005
	Condiment Junkie	67.76	<.0005
	Kassof	38.96	<.0005
	Wang	21.84	<.0005
	Deng	15.44	<.0005
	Knoeferle	4.64	.20
Sweet	Mesz–Makea	8.24	.041
	Mesz–Beethoven	38.96	<.0005
	Condiment Junkie	156.08	<.0005
	Kassof	2.16	.54
	Reinoso Carvalho	68.88	<.0005
	Deng	218.96	<.0005
	Knoeferle	104.56	<.0005

To check that there was no response bias from the participants (i.e., there were not some taste words that were simply chosen more frequently than others), we computed the total number of responses given to each taste word. There were a total of 595 bitter responses, 587 salty responses, 606 sour responses, and 612 sweet responses. A chi-square test of goodness of fit was calculated and we did not find a distribution that was significantly different from chance (χ^2^ (3, 2400) = 0.62, *p* = .89). In other words, we did not find any response bias from the participants (Erlebacher & Sekuler, 1971).

Out of 100 participants, 29 identified themselves as having no particular musical expertise, 51 identified themselves as amateurs, 17 identified as intermediates, and 3 as advanced. The participants were assigned to two groups, those with no expertise (29 people) and those with some expertise (71 people). For each soundtrack, we performed a chi-square test of independence to determine whether there was an association between musical expertise and choosing the intended taste word to match the soundtracks (see Appendix B). Did musical expertise contribute to our participants’ ability to match tastes? Out of 24 soundtracks, only Mesz’s sweet Makea soundtrack was found to have a different distribution of right/wrong choices depending on the self-reported musical expertise of the participant. In particular, a higher percentage of those participants without musical expertise chose to match the soundtrack with sweetness than participants with musical expertise.

**Figure 3.**
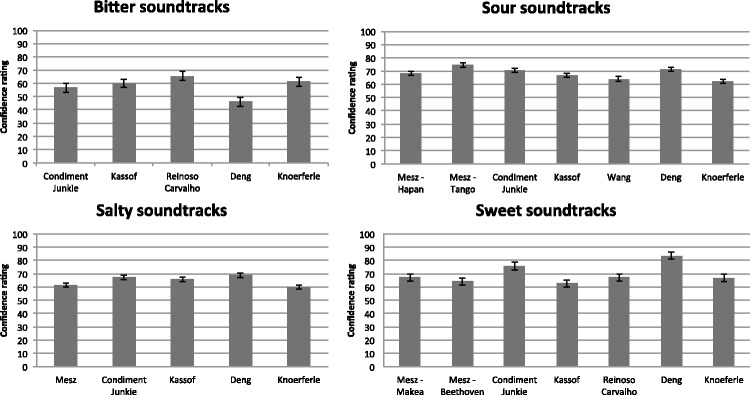
Confidence ratings of taste matches for all of the soundtracks, organized by each taste that the soundtracks were designed to evoke in Experiment 1. Confidence ratings were given on a scale from 0 to 100. For each soundtrack, the average confidence ratings over all taste matches are shown. The error bars represent the standard error of means.

The confidence ratings for each soundtrack are shown in Figure 3. Repeated measures analysis of variance (ANOVA) testing was performed within each group of soundtracks (categorized by taste) to determine if there are significant differences in confidence ratings between soundtracks. We found significant differences in confidence ratings between soundtracks in each group. For the bitter soundtracks, *F*(3.75, 371.0) = 14.24, η^2 ^= .126, *p* < .0005; for the salty soundtracks, *F*(4, 396) = 5.95, η^2 ^= .057, *p* < .0005; for the sour soundtracks, *F*(6, 594) = 8.16, η^2 ^= .076, *p* < .0005; and for the sweet soundtracks, *F*(6, 594) = 8.16, η^2 ^= .076, *p* < .0005.

More specifically, for bitter soundtracks, participants were the least confident about their taste word choice for the Deng bitter soundtrack compared with all other bitter soundtracks. The participants were also less confident about the Condiment Junkie bitter soundtrack as compared with the Reinoso Carvalho bitter soundtrack (*p* < .005 for all comparisons). Ironically, the two bitter soundtracks that participants were the least confident about—Deng and Condiment Junkie—were also the only soundtracks where bitter was chosen by the highest percentage of participants. For salty soundtracks, participants were significantly more confident about the Deng salty soundtrack than for Mesz and Knoeferle, and more confident about the Condiment Junkie soundtrack than for Knoeferle (*p* < .05 for all comparisons). This together with the fact that the Deng salty soundtrack was the most matched with salty validates the effectiveness of the soundtrack in evoking salty tastes. For sour soundtracks, participants were significantly more confident about their taste matches for the Mesz–Tango soundtrack than for the Kassof, Wang, and Knoeferle soundtracks; participants were also more confident about the Deng soundtrack as compared with the Wang and Knoeferle soundtracks; finally, they were more confident about the Condiment Junkie soundtrack than the Knoeferle soundtrack (*p* < .05 for all comparisons). The Mesz–Tango soundtrack was also the one that was most frequently matched with sourness, followed by the Deng soundtrack. For sweet soundtracks, participants were significantly more confident about the Deng soundtrack than all other sweet soundtracks. In addition, they were more confident about the Condiment Junkie soundtrack than all other sweet soundtracks except for Deng’s (*p* < .005 for all comparisons). As in the sour soundtrack case, the Deng soundtrack was also the soundtrack that was matched most with sweetness, followed by Condiment Junkie’s soundtrack. All post-hoc comparisons used Bonferroni corrections.

In summary, for the salty, sour, and sweet soundtracks, the soundtracks that were most frequently matched with the tastes that had been intended by the composers were also those where the participants were most confident about their choices. The only exception was for the bitter soundtracks.

**Figure 4.**
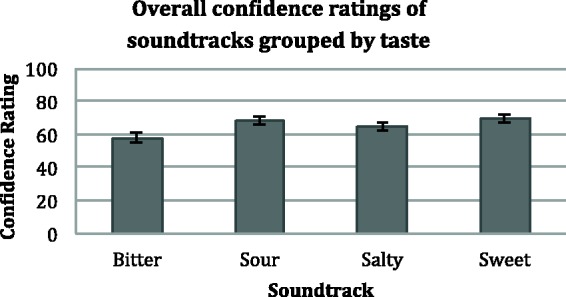
Overall confidence ratings for the various soundtracks in Experiment 1. Participants rated their taste match choices on a scale of 0 to 100 for each soundtrack. The error bars represent the standard error of means.

On average, the confidence levels for the bitter soundtracks were 57.80, *SD* = 17.57, for the salty soundtracks: *M* = 64.50, *SD* = 17.03, for the sour soundtracks: *M* = 68.18, *SD* = 16.18, and for the sweet soundtracks: *M* = 69.53, *SD* = 15.78 (see Figure 4). A repeated measures analysis of variance with Huynh-Feldt corrections was performed on the average of the confidence ratings, where significant differences were found between the confidence ratings *F*(2.59, 297) = 51.01, *p* < .0005, η^2 ^= 0.34. Specifically, pairwise comparisons with Bonferroni corrections revealed that the confidence ratings for the bitter soundtracks were significantly lower than for all of the other soundtracks (*p* < .0005 for all comparisons), the confidence ratings for the salty soundtracks were significantly higher than for the bitter soundtracks (*p* < .0005) but lower than for the sweet (*p* < .0005) and sour (*p* = .001) soundtracks, and confidence ratings for sour and sweet soundtracks were not significantly different from each other, but both were significantly higher than for bitter and salty.

## Discussion

Overall, based on the taste-matching and confidence rating data, the results of the present study demonstrate that out of 24 soundtracks (five bitter, five salty, seven sour, and seven sweet) the sweet soundtracks most effectively evoked the tastes intended by the composer (i.e., sweetness), while the bitter soundtracks were the least effective. Specifically, 56.9% of the participants’ responses were correct for sweet soundtracks, and confidence ratings for sweet soundtracks were significantly higher (*M* = 69.53, *SD* = 15.78) than for bitter and salty soundtracks; on the other hand, 31.4% of participants’ responses were correct for bitter soundtracks, and confidence ratings were significantly lower for bitter soundtracks (*M* = 57.80, *SD* = 17.57) than for all other soundtracks. The reason why our participants found it easiest to match soundtracks to sweetness is possibly because, out of all the tastes, people typically like sweetness most (Robin, Rousmans, Dittmar, & Vernet-Maury, 2003). Unlike the other tastes, sweetness is almost always pleasant, so perhaps there is a straightforward association here for participants with music and soundscapes that they find pleasant, whereas all the other tastes become associated with unpleasant music. In addition, it is perhaps worth noting that sweetness is the only taste term that is also used in musical notation, that is, *dolce*, meaning to play in a gentle, sweet style (see Fallows, 2015). Therefore participants—especially those with musical training—might more readily associate music with sweetness (though, it should be said, we did not observe this in the present study). Furthermore, our finding that sweetness somehow stands out from other tastes does not only apply to sound-taste correspondences; in shape-taste matching studies, sweetness is consistently matched to round shapes, whereas all of the other tastes are consistently matched to shapes that are more or less angular (Velasco et al., 2015, 2016).

Looking more closely at Table 1, we can try to deduce why certain soundtracks were more highly matched with tastes other than the one that had been intended by the composer.^2^ For instance, the Reinoso Carvalho bitter soundtrack was the only nonlegato bitter soundtrack and it was matched mostly with salty or sweet tastes. Similarly, Mesz–Makea, the only nonconsonant sweet soundtrack, was rated as more bitter than sweet.

Given the above-chance results of participants in matching soundtracks to their intended tastes, we were curious whether emotion played a role in mediating the associations between the soundtracks and tastes, and whether the degree and type (pleasantness or arousal) of emotion mediation differed for each basic taste. These questions are addressed in Experiment 2.

## Experiment 2

### Methods

#### Participants

Fifty participants (21 women, 29 men) aged between 20 and 64 years (*M* = 35.71, *SD* = 11.30) took part in the study. The participants gave their informed consent and reported no cold or other impairment of their senses of smell, taste, or hearing. The participants were recruited from Amazon’s Mechanical Turk (Buhrmester et al., 2011; Crump et al., 2013). The experiment was approved by the central university research ethics committee of Oxford University (MSD-IDREC-C1-2014-205).

#### Procedure

The experiment was programmed on the Xperiment experiment-design and hosting platform. Before the actual study began, the participants specified their gender, age, country of origin, and self-rated their musical expertise levels (the choices were none, amateur, intermediate, or advanced). The participants had to listen to all 24 soundtracks in a random order. After each soundtrack, the participants had to rate how pleasant and how energizing/exciting they found the soundtrack, each on a scale from 0 to 100. At the end of the test, participants were also asked how pleasant and how energizing/exciting they found bitter/salty/sour/sweet-tasting foods.

The study lasted for approximately 10 minutes, and the participants were paid $1.20 USD for taking part in the study.

### Results

**Figure 5.**
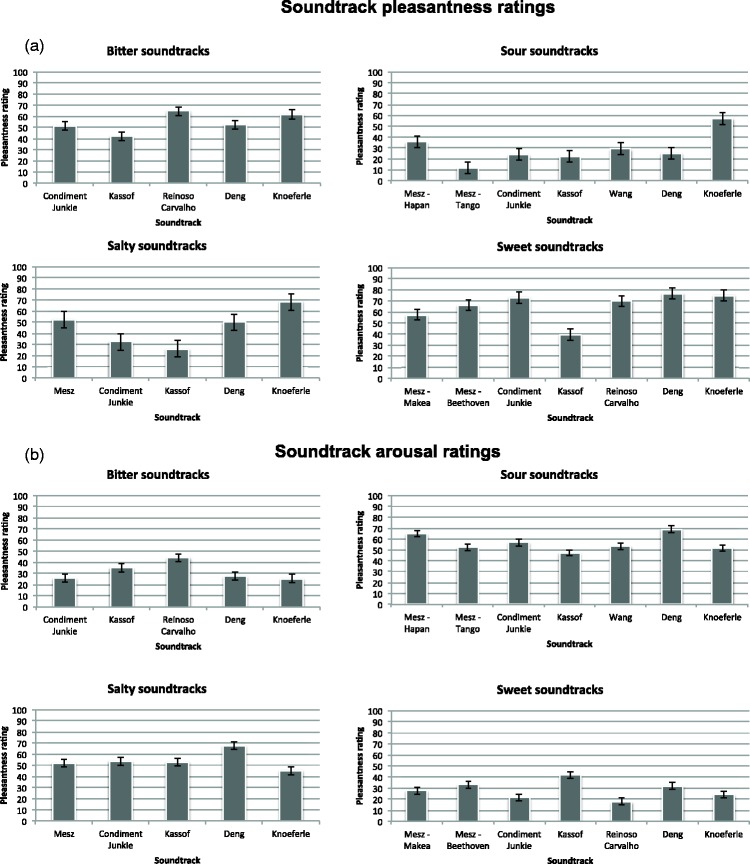
Mean pleasantness (a) and arousal (b) rating for each soundtrack, organized by each taste that the soundtracks were designed to evoke in Experiment 2. Pleasantness and arousal are each rated on a scale of 0 to 100. The error bars represent the standard error of means.

**Figure 6.**
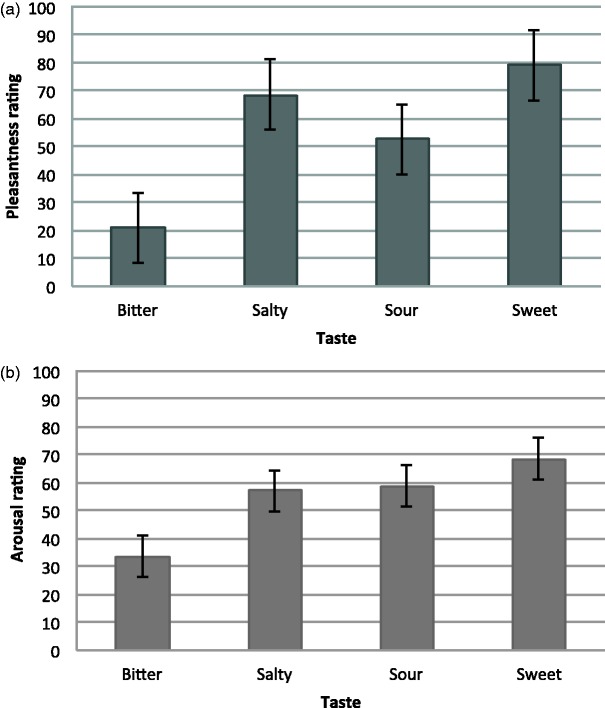
Mean pleasantness (a) and arousal (b) rating for each taste word, each on a scale of 0 to 100 in Experiment 2. The error bars represent the standard error of means.

The mean pleasantness and arousal ratings for each soundtrack are shown in Figure 5. The mean pleasantness and arousal ratings for foods of a given taste are shown in Figure 6.

To analyze the role of pleasantness and arousal in mediating the matching between soundtracks and a given taste (*T*), we used the method documented in Schifferstein and Tanudjaja (2004). For each of the 24 × 4 = 96 possible soundtrack-taste combinations, we calculated the absolute difference between the mean pleasantness rating of the soundtrack and the mean pleasantness rating of the given taste. We then calculated the correlation between this soundtrack-taste difference and the % of responses that matched the given taste to the soundtrack. If the pleasantness of a taste *T* and of a soundtrack *S* influenced the way people matched *T* and *S*, then we would expect the measure of pleasantness difference between taste *T* and soundtrack *S* to be negatively related to the % responses that match taste *T* to soundtrack *S*. The same procedure was repeated for arousal.

**Figure 7.**
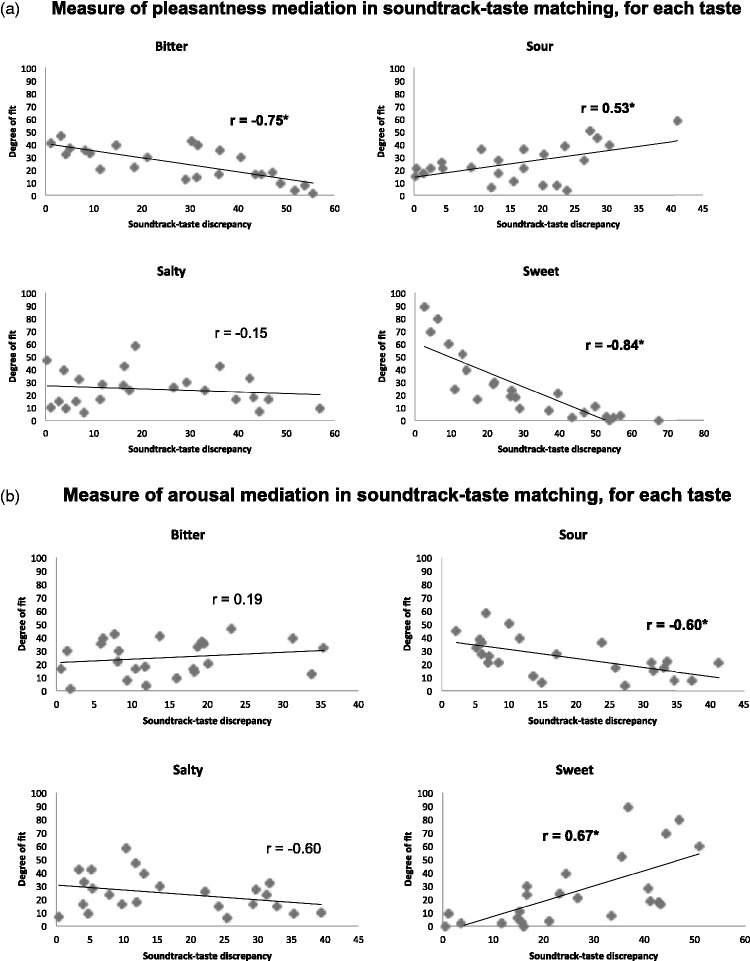
A measure of the degree of pleasantness (a) and arousal (b) mediation in soundtrack-taste matching, shown as the relationship between soundtrack-taste discrepancy from Experiment 2 (in terms of differences in pleasantness/arousal ratings of a given soundtrack-taste pair) and the degree of fit between each soundtrack and a given taste (represented by the % of responses to the soundtrack-taste matching question for a given taste from Experiment 1). The Pearson correlation coefficient is shown on each plot along with a linear regression trend line. Asterisks after the correlation coefficient indicate statistical significance (*p* < .05).

For pleasantness, Pearson correlation coefficients of −0.85 (*p* < .0005) were observed for bitterness, −0.15 (*p* = .48) for saltiness, 0.53 (*p* = .008) for sourness, and −0.84 (*p* < .0005) for sweetness (see Figure 7(a) for plots). This suggests that pleasantness partly mediates the soundtrack-taste correspondence for bitterness and sweetness.

As far as arousal was concerned, Pearson correlation coefficients of 0.19 (*p* = .39) were obtained for bitterness, −0.323 (*p* = .12) for saltiness, −0.60 (*p* = .002) for sourness, and 0.67 (*p* < .0005) for sweetness (see Figure 7(b) for plots). This suggests that arousal partly mediates the soundtrack-taste correspondence for sourness.

Furthermore, we calculated partial correlation coefficients to assess whether the effects for pleasantness and arousal are independent. The coefficient for pleasantness remained significant for the soundtrack-bitter taste (*r* = −.78, *p* < .0005) and soundtrack-sweet taste (*r* = −.72, *p* < .0005) matches when it was controlled for the effect of arousal. Similarly, the coefficient for arousal (*r* = −.53, *p* = .009) remained significant for soundtrack-sour taste matches when the effect of pleasantness was controlled for.

## General Discussion

The results of Experiments 1 and 2 demonstrate that participants performed at an above-chance level in matching soundtracks to their intended tastes, and that pleasantness and arousal partially mediated this sound-taste association. So then, to summarize, what are the factors governing people’s associations of these soundtracks with basic tastes (taste words)? First and foremost, the studies where many of the soundtracks originated have already demonstrated associations between auditory parameters and tastes (Bronner, Frieler, Bruhn, Hirt, & Piper, 2012; Crisinel & Spence, 2009, 2010; Mesz et al., 2011; see Knoferle & Spence, 2012, for a review). For instance, bitterness appears to be associated with sounds that are low in pitch (Crisinel & Spence, 2009; Knoeferle et al., 2015; Mesz et al., 2011); interestingly, Condiment Junkie’s bitter soundtrack, the most effective bitter soundtrack, also had the lowest pitch of all the bitter soundtracks (in fact, it had the lowest pitch of all 24 of the soundtracks). It has also been shown that the sound of the piano is most closely matched with sweetness (Crisinel & Spence, 2010); the Deng sweet soundtrack, which was matched with the word sweet by 89% of participants, makes liberal use of consonant high piano notes in the composition.

In addition, an important role of emotional mediation was also highlighted. As shown by the results of Experiment 2, pleasantness partly mediates soundtrack-taste matches for sweetness and bitterness, while arousal partly mediates soundtrack-taste matches for sourness. Whether intentional or not, the soundtrack designers seem to have captured some emotional aspects of tastes in their soundtracks. For instance, sweet soundtracks tend to be pleasant, whereas bitter soundtracks tend to be unpleasant. As for sour soundtracks, they tend to be the most arousing/exciting of all the soundtracks.

Lastly, some of the soundtracks—such as those by Condiment Junkie and Deng—also used semantic associations in the form of salt shaker sounds in the salty soundtrack (in fact, the prominent salt shaker sound in the background of Deng’s soundtrack might have been the reason why it had the highest matching rate—58%—out of all the salty soundtracks). In light of the emotion mediation results from Experiment 2, it is interesting to note that saltiness is the only taste whose crossmodal correspondence with the soundtracks does not appear to be mediated by pleasantness or arousal. Perhaps this is why the use of straight-forward semantic associations was so effective for the salty soundtracks. On the other hand, relying on semantic associations obscures crossmodal correspondences that are yet to be discovered between sounds and saltiness. In the future, it may be useful for researchers and designers to focus their energy on a creating a salty soundtrack which does not involve salt shaker sounds!

Incidentally, it is interesting to note that for three out of the seven sour soundtracks, bitter and sour constituted the majority of the responses (for instance, Mesz–tango had 58 responses for sour and 33 responses for bitter, Deng’s sour soundtrack had 50 responses for sour and 32 for bitter. See also Figures 1 and 2). Relevant here are previous findings that people tend to confuse *sour* and *bitter* more than the other tastes (e.g., Meiselman & Dzendolet, 1967; O’Mahony, Goldenberg, Stedmon, & Alford, 1979). So, for instance, O’Mahony et al. found that when participants were asked to apply taste adjectives (sweet, sour, salty, and bitter) to actual taste solutions, the most common error was calling the citric acid solution *bitter* instead of *sour*. In the context of the present study, the sour soundtracks might have evoked the idea or sensation of sourness, but for those participants who associate the feeling of sourness with the word *bitter*, they might have chosen to match the soundtracks with bitterness instead of sourness. A follow-up experiment in which the participants are asked to match soundtracks with unlabeled taste solutions as opposed to taste words would verify whether the sour-bitter confusion contributed to many sour soundtracks being labeled as bitter.

To be fair, some of the soundtracks—such as those created by Knoeferle and Kassof—were designed as a set in which some auditory parameters varied over the same melody (while others remained constant). This may not be as effective when participants are asked to match the sounds to tastes without having the relevant context (in Knoeferle et al.’s, 2015, study, e.g., the participants heard all four sounds and were asked to make a one-to-one mapping with the four taste words), which might account for why we did not observe a distribution of responses that was significantly different from chance for some of the soundtracks by Knoeferle and Kassof.

In addition, a possible limiting factor in the design of the present study was the fact that we only used a 15-second excerpt from each soundtrack. One could argue that, because we did not use the soundtracks in their entirety, the excerpts that were sampled might not have been the ideal (most informative) sections for matching with the desired tastes. Our decision to limit the length of each sound sample was designed to avoid participant fatigue. As all the soundtracks were looping and fairly uniform in texture, it should not make a difference where we sampled the music. To be sure, this is quite different from traditional music where there is a defined beginning, middle, and end. For taste, music is usually atmospheric and hence has more of a repetitive nature.We thought the first 15 seconds should be sufficient to evoke the desired tastes, especially for the soundtracks used in experiments involving tasting real food products (soundtracks by Condiment Junkie, Mesz, Reinoso Carvalho, and Wang), where participants should feel the effect of matched taste as soon as possible.

Lastly, the present results highlighted an example of the role that musical expertise can play in matching music with tastes. For Mesz’s sweet Makea piece, those with no musical experience were significantly more likely to match it to sweetness than those with musical experience (for whom bitterness was the most common choice). This was potentially because Makea features dissonant chords and high pitched piano instrumentation; as musical novices tend to focus on timbre while those with musical experience tend to focus on melody and harmony (Wolpert, 1990), perhaps novices matched the high-pitched piano sounds to sweetness, while more experienced listeners matched the dissonant chords to bitterness. This highlights the importance for future compositions to have consistent musical features across all levels of music cognition.

The recent explosion of studies and performances that have attempted to bring together sound and taste demonstrate the level of interest in *sonic seasoning*, by which sound can be used to alter the taste of a dish. This study contributes to the effort by validating which soundtracks that people can reliably match to tastes. Once these correspondences are found, one can then study any perceptual effects of these soundtracks on the taste of real foodstuffs. From the soundtracks studied here, the earlier versions of bitter and sweet soundtracks by Condiment Junkie have been shown to significantly change people’s ratings of toffee on a bitter-sweet scale (Crisinel et al., 2012). Meanwhile, the bitter and sweet soundtracks generated by Reinoso Carvalho were used in a chocolate-tasting study in which the sweet soundtrack significantly changed people’s rating of bitter chocolate on a bitter-sweet scale (Reinoso Carvalho, Van Ee, Rychtarikova, Touhafi, Steenhaut, Persoone, Spence, & Leman, 2015). It remains to be seen whether the most effective soundtracks here, such as Mesz’ sour tango soundtrack or Deng’s sweet soundtrack, can lead to measurable taste modifications.

Surely, in the years to come, the demand for future research into sound-taste correspondences will only increase, especially in the light of recent studies showing how customers are willing to pay significantly more for food/drink when it is accompanied by matching multisensory stimuli than without (Michel, Velasco, Gatti, & Spence, 2014; Reinoso Carvalho, Van Ee, Rychtarikova, Touhafi, Steenhaut, Persoone, & Spence, 2015). The hope is that this type of comparative study will help guide design decisions for soundtracks and experiments as more people explore this intriguing area between sound and taste.

## Notes

1 Xperiment is an open-source research platform that offers experiment development, hosting, and data collection on a variety of devices (http://www.xperiment.mobi). It offers experimenters a script-based system to create and upload experiments and publishes directly to crowd sourcing sites (Woods, Velasco, Levitan, Wan, & Spence, 2015).

2 Three sour soundtracks had nonrandomly distributed matches but participants matched them slightly more with bitter than sour taste words. The sour-bitter conundrum is addressed further in general discussions.

## Appendix A

Taste-matching counts from the soundtrack-taste matching task: For each soundtrack, the participants had to choose one taste word (bitter, salty, sour, or sweet) that best matched the soundtrack. The tables below highlight the number of responses per taste word that each soundtrack received.

**Table:**

Bitter soundtracks	Condiment Junkie	Kassof	Reinoso Carvalho	Deng	Knoeferle
**Bitter**	**42**	**30**	**16**	**39**	**30**
Salty	23	26	39	27	32
Sour	17	36	6	15	22
Sweet	18	8	39	19	16

**Table:**

Salty soundtracks	Mesz	Condiment Junkie	Kassof	Deng	Knoeferle
**Salty**	**42**	**42**	**33**	**58**	**47**
Sour	21	32	27	21	11
Sweet	23	6	3	9	24
Bitter	14	20	37	12	18

**Table:**

Sour soundtracks	Mesz–Hapan	Mesz–Tango	Condiment Junkie	Kassof	Wang	Deng	Knoeferle
**Sour**	**36**	**58**	**45**	**39**	**38**	**50**	**26**
Bitter	39	33	46	41	35	32	16
Salty	23	9	7	16	16	18	28
Sweet	2	0	2	4	11	0	30

**Table:**

Sweet soundtracks	Mesz–Makea	Mesz–Beethoven	Condiment Junkie	Kassof	Reinoso Carvalho	Deng	Knoeferle
**Sweet**	**28**	**52**	**79**	**21**	**60**	**89**	**69**
Salty	16	15	9	30	10	6	15
Sour	21	17	8	27	21	4	8
Bitter	35	16	4	22	9	1	8

### Appendix B

Taste-matching counts from soundtrack-taste matching task, sorted by musical expertise.

**Table:**

Bitter soundtracks						
Experience		Condiment Junkie	Kassof	Reinoso Carvalho	Deng	Knoeferle
None	Bitter	14	7	4	10	12
	Other	15	22	25	19	17
Some	Bitter	28	23	12	29	18
	Other	43	48	59	42	53

**Table:**

Salty soundtracks						
Experience		Mesz	Condiment Junkie	Kassof	Deng	Knoeferle
None	Salty	11	10	8	16	17
	Other	18	19	21	13	12
Some	Salty	31	32	25	42	30
	Other	40	39	46	29	41

**Table:**

Sour soundtracks								
Experience		Mesz–Hapan	Mesz–Tango	Condiment Junkie	Kassof	Wang	Deng	Knoeferle
None	Sour	12	17	13	10	12	13	4
	Other	17	12	16	19	17	16	25
Some	Sour	24	41	32	29	26	37	22
	Other	47	30	39	42	45	34	49

**Table:**

Sweet soundtracks								
Experience		Mesz–Makea	Mesz– Beethoven	Condiment Junkie	Kassof	Reinoso Carvalho	Deng	Knoeferle
None	Sweet	14	13	24	5	15	27	19
	Other	15	16	5	24	14	2	10
Some	Sweet	14	39	55	16	45	62	50
	Other	57	32	16	55	26	9	21
